# ATPPred: Prediction of Plant ATP-Binding Proteins Using ESM2 and 1D Convolution

**DOI:** 10.3390/ijms27198566

**Published:** 2026-09-25

**Authors:** Hong-Qi Zhang, Zi-Xuan Zhang, Yi-Xuan Qi, Cheng Chen, Jian Huang, Hao Lin, Ke-Jun Deng, Juan Feng

**Affiliations:** Center for Informational Biology, School of Life Science and Technology, University of Electronic Science and Technology of China, Chengdu 610054, China; hqzhang215@163.com (H.-Q.Z.); zhangzixuan_02@163.com (Z.-X.Z.); qiyixuan1024@163.com (Y.-X.Q.); 18976949153@163.com (C.C.); hj@uestc.edu.cn (J.H.); hlin@uestc.edu.cn (H.L.)

**Keywords:** evolutionary scale modeling, deep learning, transformer, plant biology, disease mechanism

## Abstract

Plant ATP-binding proteins play a central role in various physiological processes in plants, including growth and development, energy metabolism, signal transduction, and environmental adaptation. Accurately identifying ATP-binding proteins from plant protein sequences is crucial for a deeper understanding of plant physiology and their applications in agricultural biotechnology. In this study, we propose ATPPred, a tool based on the protein large language model Evolutionary Scale Modeling 2 (ESM2) and a 1-dimensional convolutional neural network for the high-precision prediction of plant ATP-binding proteins. The tool extracts global features of plant ATP-binding proteins using ESM2 and then further extracts feature information with the 1-dimensional convolutional neural network. Comprehensive performance metrics demonstrate ATPPred’s competitive prediction capability. A systematic evaluation of the impact of protein sequence similarity thresholds on ATPPred yielded an average AUC ranging from 0.9191 ± 0.0161 to 0.9669 ± 0.0030 across thresholds from 0.4 to 0.9, highlighting its robustness. Cross-dataset benchmarking of ATPPred further validated the effectiveness of our framework, showcasing superior performance compared to existing methods. In summary, ATPPred provides a feasible computational approach for sequence-based prediction of plant ATP-binding proteins and may serve as a useful auxiliary tool for the preliminary screening and prioritization of candidate ATP-binding proteins.

## 1. Introduction

Adenosine 5′-triphosphate (ATP), the central molecule in cellular energy metabolism, plays a pivotal role in various physiological processes of plants, including growth and development [1], nutrient absorption [2], signal transduction [3], stress responses [4], and the regulation of secondary metabolism [5]. Its biological functions are primarily mediated through phosphate group transfer reactions and specific protein target interactions [6]. Recent studies have demonstrated that the S-acylation modification of plant immune receptor P2K1 (which also functions as an ATP receptor) suppresses immune responses, thereby modulating plant defense mechanisms against environmental stimuli [7]. Mevalonate kinase (MVK) interacts with and phosphorylates P2K1 receptors, directly influencing key metabolic pathways in plant cells [8]. Beyond these established roles, ATP-binding proteins also impact plant physiology through diverse mechanisms. For instance, a novel ATP-binding cassette (ABC) transporter has been identified to participate in aluminum detoxification in plants [9]. Another ABC transporter protein—ABCB19, a brassinosteroid membrane transporter—plays a critical role in plant responses to abiotic stress [10,11,12], underscoring the multifaceted roles of ATP-binding proteins not only in energy metabolism but also in environmental stress adaptation. These findings collectively highlight the essential contributions of ATP-binding proteins to plant metabolic regulation and adaptive responses. Therefore, developing methods for predicting plant ATP-binding proteins holds significant theoretical implications for elucidating plant energy networks and has practical applications in targeted drug design and agricultural biotechnology innovation.

Experimental approaches such as mutant screening and gene mapping can provide high-confidence functional evidence for proteins [13,14], though such approaches are inherently associated with high costs, long experimental cycles, and limitations in high-throughput processing. With the rapid advancement of bioinformatics [15,16], computational models for ATP-binding prediction have emerged as promising solutions to address these bottlenecks [17,18,19,20,21,22]. In 2014, researchers built a hybrid feature classifier using the LIBSVM toolkit that does not rely on specific functional motifs [23,24,25,26]. By integrating features derived from amino acid sequences with dipeptide composition information [27,28], the model achieved high-precision generalized predictions for ATP-binding proteins [29]. In 2020, a random forest classifier enhanced the generalization performance of the identification of ABC transporters through 188-dimensional feature encoding of sequence information and physicochemical properties [30].

Despite considerable efforts by researchers to develop high-performance and reliable ATP-binding protein prediction models, existing methods still face several technical bottlenecks and challenges: (1) The performance of current models still has room for improvement. (2) No studies have explored the application of large language models in the prediction model of plant ATP-binding proteins. (3) While protein sequence similarity still poses a significant challenge to the model predictions, existing research has not systematically evaluated how changes in similarity thresholds affect the performance of plant ATP-binding protein prediction models.

We present ATPPred, a precise prediction tool for plant ATP-binding proteins, combining Evolutionary Scale Modeling 2 (ESM2) and the 1-dimensional convolutional neural network (1D CNN). It achieved strong performance in metrics such as accuracy (ACC), Matthews correlation coefficient (MCC), F1 score (F1), and area under the receiver operating characteristic curve (AUC). Evaluations across varying protein sequence similarity thresholds confirmed that ATPPred maintains strong performance under different similarity conditions, demonstrating high robustness. Moreover, our framework’s excellent performance on cross-dataset benchmarking validates its applicability in predicting ATP-binding proteins on different datasets. Therefore, ATPPred may serve as a computational tool for the preliminary identification and prioritization of candidate plant ATP-binding protein sequences, providing a reference for subsequent functional genomics studies in plants.

## 2. Results

### 2.1. Validation of the ATPPred Model

In this study, we developed the ATPPred prediction model with high representational capability by leveraging the global features of protein sequences captured by ESM2 and further extracting feature information through the 1D CNN. Five-fold cross-validation showed that the model exhibited strong generalization performance on the test set, with all metrics reaching high levels. Specifically, the model achieved an ACC of 0.9190 ± 0.0058, Rec of 0.8738 ± 0.0185, Pre of 0.9615 ± 0.0241, AUC of 0.9669 ± 0.0030, MCC of 0.8421 ± 0.0129, F1 of 0.9152 ± 0.0053, SP of 0.9641 ± 0.0246, and AUPRC of 0.9744 ± 0.0014 (Figure 1A, Table 1). Additionally, a 10-fold cross-validation was performed, yielding an ACC of 0.9226 ± 0.0125, an AUC of 0.9691 ± 0.0046, an MCC of 0.8494 ± 0.0214, and an AUPRC of 0.9760 ± 0.0031 (Table S1). These results demonstrate that ATPPred effectively integrates multi-scale biological features, enabling accurate prediction of plant ATP-binding proteins. Moreover, the model exhibited highly stable predictive performance across different sub-dataset splits in the five-fold cross-validation, further validating its strong robustness for plant ATP-binding protein identification tasks (Figure 1B–D, Table 1). Furthermore, multi-round random sampling evaluations of the negative dataset confirmed that the model’s strong predictive performance maintains high reliability and is independent of specific negative subset selections (Table S2).

### 2.2. Impact of Different Negative Sampling Strategies on Model Performance

To validate the model’s robustness and generalization ability under different negative sample construction methods, we kept the positive samples unchanged while adopting two random negative sampling strategies based on amino acid sequence length distribution (Length-matched) and species distribution (Species-matched), respectively, and evaluated various performance metrics of the model through a stringent five-fold cross-validation framework. Experimental results demonstrate that under both negative sampling strategies, ATPPred exhibits strong and stable predictive performance. Specifically, under the length-matched strategy, the model achieved an ACC of 0.8804 ± 0.0167, an AUC of 0.9483 ± 0.0080, and an MCC of 0.7620 ± 0.0341. Under the species-matched strategy, the overall performance remained highly consistent, with an ACC of 0.8931 ± 0.0134, an AUC of 0.9545 ± 0.0121, and an MCC of 0.7885 ± 0.0267 (Figure S1, Table S3). These comparative results fully confirm that despite the differences in negative sample construction benchmarks, core evaluation metrics are maintained at high levels with small standard deviations. This indicates that ATPPred possesses strong robustness, effectively avoiding overfitting or dramatic performance fluctuations caused by negative sampling bias.

### 2.3. Ablation Experiment Validation

To comprehensively validate the effectiveness of each core component within the ATPPred framework, we conducted a series of ablation studies and comparative analyses under identical five-fold cross-validation frameworks and hyperparameter configurations. Specifically, we evaluated three variant models: ESM + MLP, which removes the 1D CNN module and directly connects to a multi-layer perceptron (MLP) to examine the enhancing effect of the 1D CNN on ESM feature learning; ATPPred (Frozen ESM), which freezes the ESM2 feature extractor (without fine-tuning) to assess the impact of pre-trained feature fine-tuning on model performance; and ATPPred (ProtBERT), which replaces the original ESM2 with another prominent protein language model, ProtBERT, to compare the capability of different pre-trained large models in ATP-binding prediction. Experimental results demonstrate that the complete ATPPred model achieves superior performance across all core evaluation metrics (Figure 2, Table 2). Taken together, these ablation and comparative experiments robustly confirm the scientific soundness and efficiency of the “fine-tuned ESM2 combined with 1D CNN” architecture in the ATPPred framework, highlighting the synergistic effects of its constituent modules in boosting predictive performance.

### 2.4. Exploring the Impact of Protein Sequence Similarity Thresholds on the Model

Protein sequence similarity thresholds serve as a key factor in the classification performance [31], with their dynamic adjustment directly impacting the precision of feature space selection. We constructed six datasets ranging from 0.4 to 0.9 and systematically explored the robustness of the model under a stringent five-fold cross-validation framework. Across all evaluated similarity thresholds, the model consistently maintained high predictive performance, with average ACC values ranging from 0.8467 to 0.9190 and average AUC values falling within the interval of 0.9191 to 0.9669 (Figure S2, Table S4). The experimental results indicate that ATPPred is capable of reliably extracting key sequence features under various similarity threshold conditions, providing strong evidence for the model’s reliability and applicability in plant ATP-binding protein classification tasks.

### 2.5. Cross-Dataset Benchmarking

To comprehensively evaluate the performance of ATPPred on different datasets, we retrained and evaluated our model on the protein dataset constructed by Hou et al. (which includes cross-species ABC transporter positive and negative samples), referred to as the Base-Data [30]. Base-Data is a unified benchmark for model comparisons in subsequent experiments, ensuring fairness and comparability in cross-model evaluations. Through rigorous 10-fold cross-validation, we systematically analyzed the performance of ATPPred on Base-Data. The experimental results show that ATPPred exhibits strong prediction performance on Base-Data, with all core evaluation metrics maintaining high levels. Specifically, the average ACC was 0.9640 ± 0.0143, Rec was 0.9637 ± 0.0179, Pre was 0.9635 ± 0.0250, AUC was 0.9776 ± 0.0135, MCC was 0.9277 ± 0.0292, F1 was 0.9634 ± 0.0166, SP was 0.9654 ± 0.0191, and AUPRC was 0.9756 ± 0.0228, respectively (Figure S3A, Table S5). Moreover, the 10-fold cross-validation ROC curve, PRC, and confusion matrix visually demonstrate ATPPred’s strong performance on the benchmark dataset (Figure S3B–D). These results demonstrate the effectiveness and robustness of our proposed framework in identifying ATP-binding proteins.

### 2.6. Comparison with a State-of-the-Art Model

To assess the competitive advantages, we compared ATPPred with a state-of-the-art model. Since the model provided by the research team behind Base-Data has not been open-sourced, we retrained ATPPred using their publicly available experimental data to ensure fairness in cross-model comparisons. Additionally, although previous investigators proposed a hybrid prediction model based on amino acid–dipeptide combinations, we were unable to include it in this comparison as their code and data were not publicly available. The experimental results show that ATPPred outperforms the benchmark model established by Base-Data in all core evaluation metrics, with the following improvements: ACC increased by 7.66%, Rec improved by 4.88%, AUC rose by 2.14%, MCC improved by 17.21%, and SP enhanced by 10.74%, respectively (Figure 3, Table 3). These results indicate that ATPPred is highly competitive in the task of ATP-binding protein prediction.

## 3. Discussion

In this study, we introduce ATPPred, a novel computational tool designed for high-precision prediction of plant ATP-binding proteins, which first utilizes ESM2 to extract sequence representations, and subsequently applies a 1D CNN to further extract feature information. Through rigorous five-fold cross-validation, ATPPred demonstrated strong generalization capability and robustness across sub-dataset splits, achieving an average ACC of 0.9190 ± 0.0058, Rec of 0.8738 ± 0.0185, Pre of 0.9615 ± 0.0241, AUC of 0.9669 ± 0.0030, MCC of 0.8421 ± 0.0129, and AUPRC of 0.9744 ± 0.0014, while evaluations across multi-round negative sampling and distinct sampling strategies consistently yielded high core metrics with minimal standard deviations, confirming that the model effectively avoids negative sampling bias and maintains high statistical reliability. Through comprehensive ablation and comparative experiments, we verified the scientific soundness of our proposed framework, as the complete ATPPred architecture consistently outperformed all variant models across all core evaluation metrics, confirming that this combination creates performance gains. We also systematically explored the effect of sequence similarity thresholds on model performance, and ATPPred maintained highly stable prediction performance across all evaluated threshold levels, with average ACC values ranging from 0.8467 to 0.9190 and average AUC values sustained between 0.9191 and 0.9669, demonstrating the model’s strong adaptability and robustness under dynamic feature space constraints. In cross-dataset benchmark testing on the Base-Data dataset under 10-fold cross-validation, ATPPred achieved an average ACC of 0.9640 ± 0.0143, Rec of 0.9637 ± 0.0179, Pre of 0.9635 ± 0.0250, AUC of 0.9776 ± 0.0135, MCC of 0.9277 ± 0.0292, and AUPRC of 0.9756 ± 0.0228, displaying clear performance advantages when compared directly with the benchmark model, improving ACC by 7.66%, Rec by 4.88%, AUC by 2.14%, MCC by 17.21%, and SP by 10.74%, which indicates that ATPPred provides an efficient and reliable computational solution for ATP-binding protein prediction tasks. To summarize, this work makes three primary contributions: first, we construct a novel dual-module fusion architecture that first utilizes ESM2 to extract sequence representations, and subsequently applies a 1D CNN to further extract feature information; second, we verify the model’s strong robustness and dynamic adaptability through comprehensive ablation studies, multi-round negative sampling, and varying sequence similarity thresholds; and third, we achieve superior performance in cross-dataset benchmarking. However, this study still has certain limitations; for instance, although we performed cross-dataset testing on Base-Data, the model has not yet been extensively evaluated on diverse independent external validation sets entirely derived from real experimental scenarios. In future work, we will focus on collecting and constructing high-quality independent external validation sets to rigorously test the model, thereby further verifying its generalization capability.

## 4. Materials and Methods

### 4.1. Data

The data were obtained from UniProt. Both the initial positive and negative samples were screened according to the following criteria: restricted to plant species, retaining only entries with protein-level evidence, and retaining only “Reviewed” entries. The initial positive samples were defined as proteins annotated as ATP-binding. The initial negative samples were defined as proteins annotated as non-ATP-binding, and protein fragments were excluded from the initial negative samples. Finally, 1525 plant ATP-binding proteins (initial positive samples) and 6924 plant non-ATP-binding proteins (initial negative samples) were obtained [32]. First, plant ATP-binding proteins and plant non-ATP-binding proteins were filtered for redundancy using the CD-HIT (version: 4.8.1) tool [33,34], with a threshold set at 0.9. After filtering, we obtained 1391 non-redundant plant ATP-binding proteins (used as positive samples) and 6056 plant non-ATP-binding proteins. From these 6056 plant non-ATP-binding proteins, we randomly selected 1391 as negative samples, resulting in a total of 2782 data points. Subsequently, the dataset was split into training and test sets at an 8:2 ratio, with the training set containing 2224 samples (1112 positive and 1112 negative) and the test set containing 558 samples (279 positive and 279 negative). We applied the same method to collect and process data with thresholds of 0.4, 0.5, 0.6, 0.7, and 0.8, yielding datasets of sizes 1346, 1636, 1920, 2210, and 2482, respectively. Additionally, we performed precise negative sampling based on the length distribution of the positive samples, ensuring that both classes remained consistent in terms of sequence length. Any positive samples that could not be successfully matched were excluded, resulting in the length-matched benchmark dataset with a sample size of 2138. Similarly, based on the species distribution characteristics, we constructed the species-matched benchmark dataset consisting of 2756 samples. Furthermore, we collected a dataset provided by Hou et al., which includes 875 ABC transporters (positive samples) and 9735 non-ABC transporters [30]. Consistent with Hou et al.’s approach, we randomly selected 875 negative samples from the 9735 non-ABC transporters [30].

### 4.2. Methods

The esm2_t6_8M_UR50D model from the ESM2 series, a large protein language model, was used for feature extraction [35]. We retained the first four layers of the ESM2 model’s Transformer module to prevent model overfitting and enhance its generalization ability, reducing model complexity and focusing on more representative features. A 1D CNN was then applied for further feature extraction, followed by fully connected layers for final task prediction (Figure 4). All parameters of the ESM2 model were trained with no layers frozen, enabling full end-to-end optimization of the entire ATPPred architecture. All experiments were conducted on an NVIDIA GeForce RTX 4090 GPU.

#### 4.2.1. ESM2

Evolutionary Scale Modeling (ESM) is a large language model based on the Transformer architecture, designed to capture evolutionary information and structure–function relationships from large-scale protein sequence data through self-supervised learning. The core idea of ESM is to adapt the masked language modeling task from natural language processing to protein sequence analysis. It predicts the identity of randomly chosen amino acids using the context of the remaining parts of the protein sequence, enabling the model to learn dependencies between amino acids. ESM2, as the second-generation model in this series, significantly improves parameter efficiency and representation power through optimizations in architecture, training strategies, and computational resources [35]. In ESM2, rotary position embedding (RoPE) was adopted to enable extrapolation beyond the training context window. The esm2_t6_8M_UR50D model used in this study contains 6 layers of Transformer modules and 8 million parameters. We reduced the complexity by retaining the first four layers of the Transformer modules to ensure better generalization. The input to the ESM2 model is a protein’s amino acid sequence, which is processed through the layers of the Transformer model to generate a representation for each amino acid. This representation captures the complex relationships between amino acids, particularly interactions between distant amino acids. We can obtain a 320-dimensional feature of the amino acid sequence for protein function prediction. The attention mechanism can effectively pass information between different amino acids using self-attention. The calculation is expressed by the following formula:

(1) $$\mathrm{Attention}\left(Q,K,V\right)=softmax\left(\frac{{QK}^{T}}{\sqrt{D_{K}}}\right)V$$

*Q*, *K*, and *V* refer to the query, key, and value matrices, respectively, with $D_{K}$ indicating the dimensionality of the vector.

#### 4.2.2. 1D CNN

1D CNN is a commonly used neural network method for feature extraction, which captures spatial or pattern features in the input data through local weighted summation [36,37,38,39]. Unlike 2D CNN, 1D CNN slides the kernel along a single dimension, progressively capturing local patterns. Convolutional layers are more effective at extracting local features from data compared to traditional fully connected layers. The number of parameters is significantly reduced due to the weight-sharing mechanism, enhancing computational efficiency and reducing the risk of overfitting. Therefore, 1D CNN was applied to the high-dimensional features extracted by ESM2 to enhance feature representation and help the model better learn meaningful patterns.

After feature extraction with ESM2, we used a two-layer 1D CNN to further abstract and extract deeper features. Let the input features be $x\in R^{n}$, where n is the dimensionality of the input features, and the convolutional kernel be $w\in R^{k}$,where k is the size of the kernel. The output $y\in R^{n-k+1}$ of the 1D convolution operation can be expressed as:

(2) $$y_{t}={\left(X*W\right)}_{t}=\sum_{i=0}^{k-1}x_{t+i}\cdot W_{i}$$

here, t represents the position of the convolution, and $w_{i}$ is the i-th element of the convolutional kernel. By sliding the convolutional kernel w over the input features x, a new feature sequence y is generated.

The first convolutional layer has 8 kernels, each with a size of 3, while the second convolutional layer has 2 kernels, each also with a size of 3. Additionally, we introduced the nonlinear activation function ReLU, with the following formula:

(3) $$ReLU\left(z\right)=max\left(0,z\right)$$

here, *z* is the input value.

### 4.3. Parameter Settings

To determine the optimal batch size, we evaluated various batch size configurations (specifically 1, 2, 4, and 8) on the training set. A batch size of 1 yielded the best performance, and the selected configuration also performed well on the test set (Table S6). Consequently, we configured the model to train for 50 epochs with an optimal batch size of 1, a learning rate of 0.003, and a dropout rate of 0.1 to mitigate overfitting. Additionally, the fully connected network consisted of two hidden layers and an output layer, with hidden dimensions of 32 and 8, respectively. The activation function was ReLU, the optimizer was SGD, and the classification threshold was set to 0.5. We did not manually truncate the protein sequences in any way. Finally, 5-fold cross-validation was employed on the training set for hyperparameter selection.

### 4.4. Evaluation Metrics

Several performance metrics were used to assess the model, including ACC, Recall (Rec), Precision (Pre), Specificity (SP), F1, and MCC [40,41,42,43]. To comprehensively analyze the model’s predictive capability, we also plotted the Receiver Operating Characteristic (ROC) curve and the Precision–Recall Curve (PRC), and calculated the Area Under the Curve (AUC and AUPRC) to measure the overall performance of the ROC and PRC, respectively [26,44,45,46,47,48,49,50,51]. The specific formulas for these metrics are as follows:

(4) $$ACC=\frac{TP+TN}{TP+FP+TN+FN}$$

(5) $$Pre=\frac{TP}{TP+FP}$$

(6) $$Rec=\frac{TP}{TP+FN}$$

(7) $$SP=\frac{TN}{TN+FP}$$

(8) $$F1=\frac{2PreRec}{Pre+Rec}$$

(9) $$MCC=\frac{TP\times TN-FP\times FN}{\sqrt{\left(TP+FP\right)\left(TN+FN\right)\left(TP+FN\right)\left(TN+FP\right)}}$$

here, *TP*, *TN*, *FP*, and *FN* represent the true positive, true negative, false positive, and false negative samples, respectively [52,53,54,55].

## 5. Conclusions

This study presents ATPPred, a novel tool that combines the ESM2 protein language model with a 1D convolutional neural network for prediction of plant ATP-binding proteins. ATPPred demonstrates superior performance across several key evaluation metrics, particularly in ACC, AUC, and MCC, outperforming the ESM-only model. Further experiments show that the ATPPred architecture exhibits excellent applicability across different datasets and robust performance under varying sequence similarity thresholds. Comparative results with an existing model highlight ATPPred’s advantages across all key evaluation metrics. In the future, we believe that ATPPred will serve as a computational approach for the preliminary identification and prioritization of candidate plant ATP-binding protein sequences, providing a reference for subsequent functional analyses and experimental validation.

## Figures and Tables

**Figure 1:**
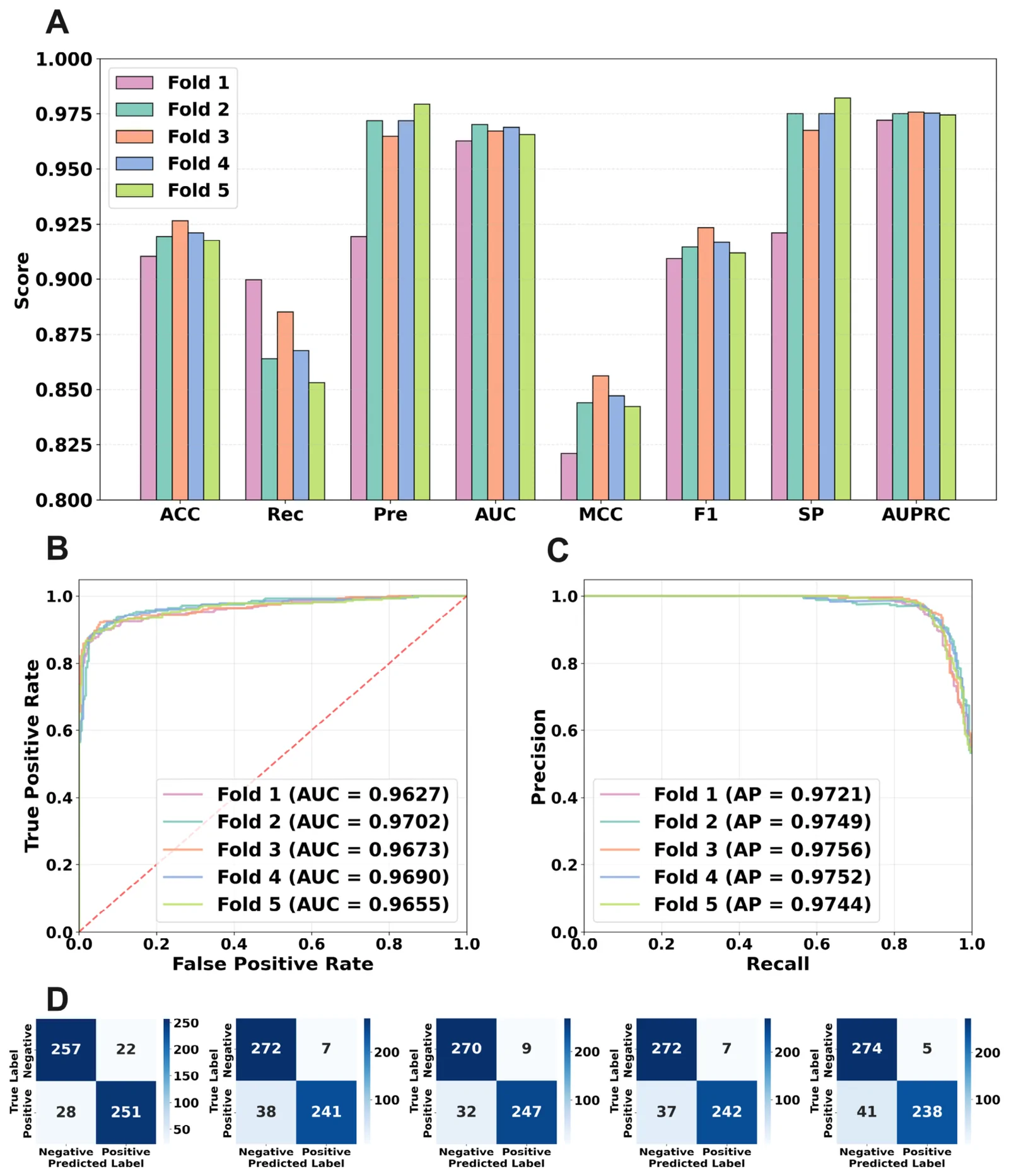
Evaluation results of ATPPred on the test set. (**A**) Performance metrics of ATPPred. (**B**) ROC curve performance of ATPPred. (**C**) PRC performance of ATPPred. (**D**) Confusion matrix of ATPPred.

**Figure 2:**
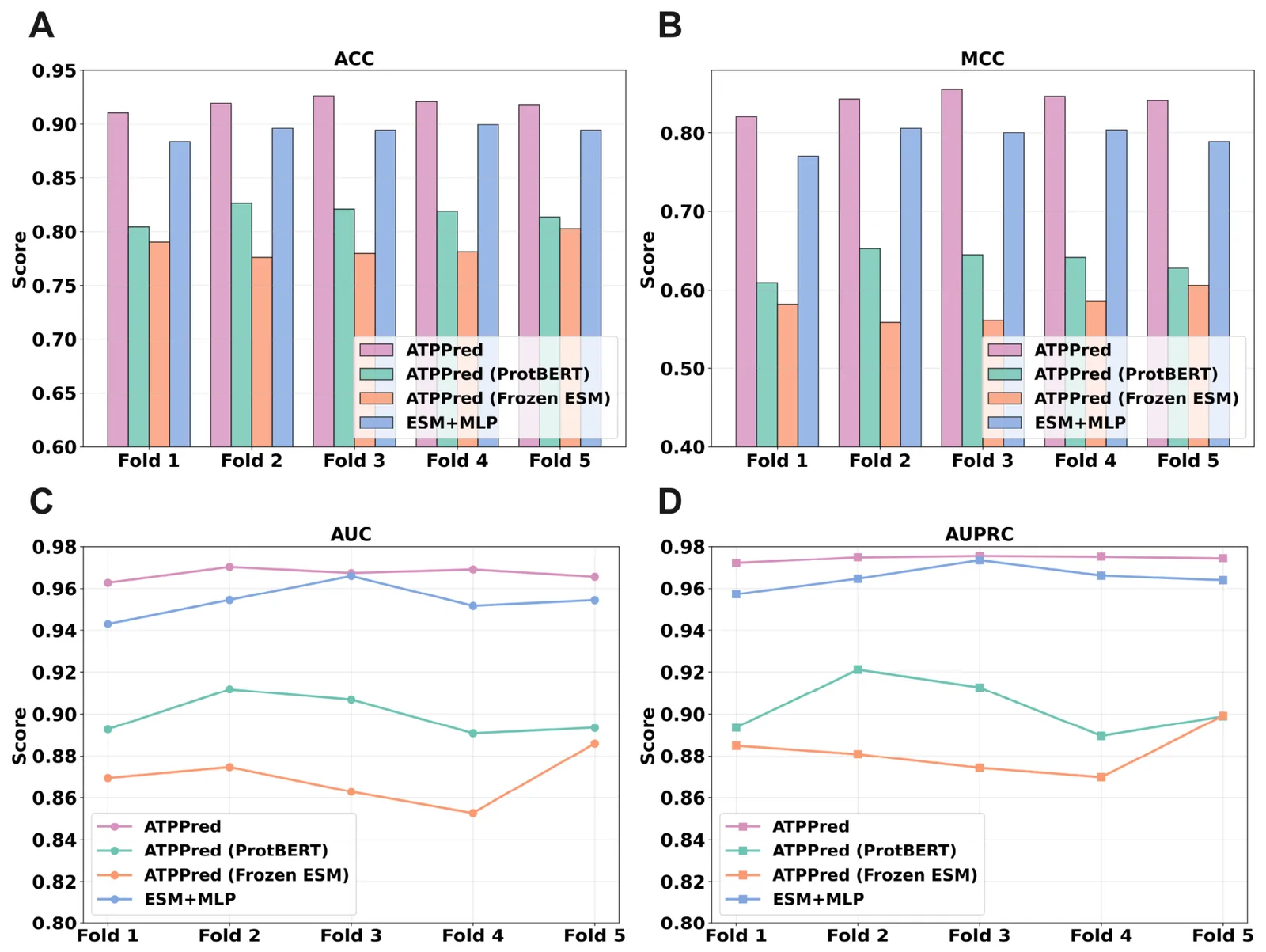
Ablation experiment results on the test set. (**A**) Comparison of ACC results. (**B**) Comparison of MCC results. (**C**) Comparison of AUC results. (**D**) Comparison of AUPRC results.

**Figure 3:**
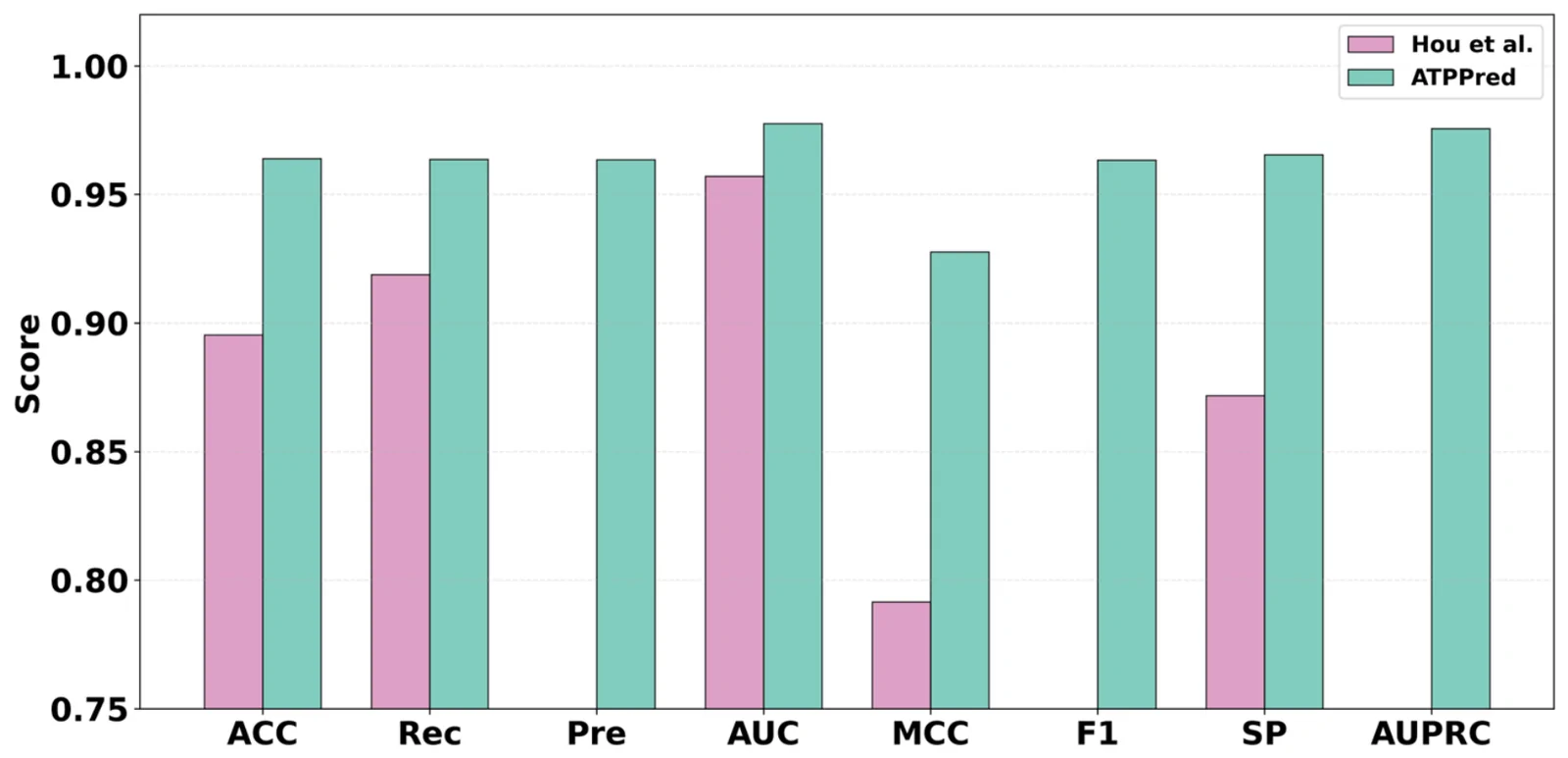
Performance comparison with Hou et al.’s method [30].

**Figure 4:**
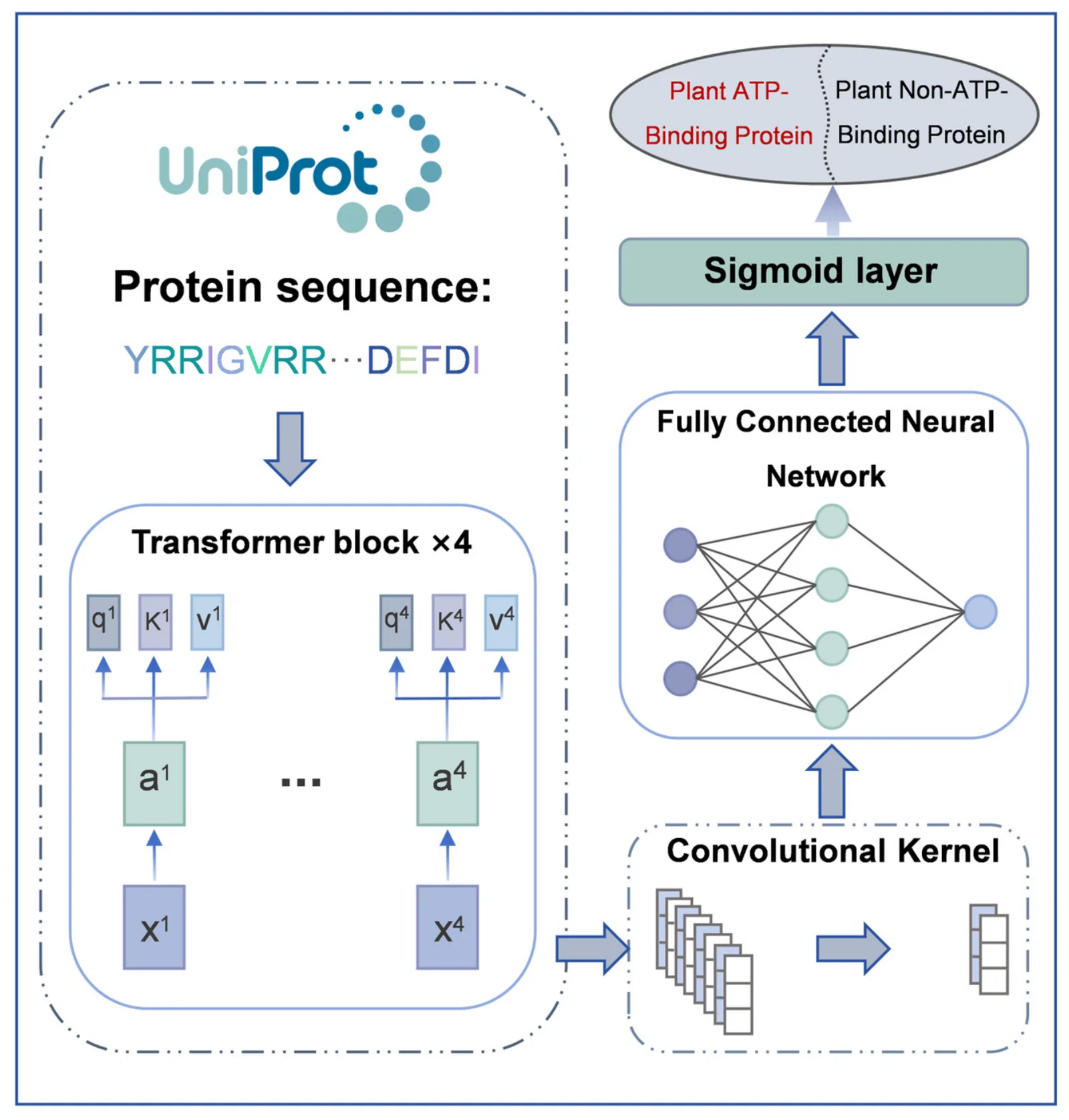
Model architecture.

**Table 1:** Detailed evaluation results of ATPPred on the test set.

Fold	ACC	Rec	Pre	AUC	MCC	F1	SP	AUPRC
Fold 1	0.9104	0.8996	0.9194	0.9627	0.8210	0.9094	0.9211	0.9721
Fold 2	0.9194	0.8638	0.9718	0.9702	0.8439	0.9146	0.9749	0.9749
Fold 3	0.9265	0.8853	0.9648	0.9673	0.8560	0.9234	0.9677	0.9756
Fold 4	0.9211	0.8674	0.9719	0.9690	0.8472	0.9167	0.9749	0.9752
Fold 5	0.9176	0.8530	0.9794	0.9655	0.8422	0.9119	0.9821	0.9744
Avg	0.9190 ± 0.0058	0.8738 ± 0.0185	0.9615 ± 0.0241	0.9669 ± 0.0030	0.8421 ± 0.0129	0.9152 ± 0.0053	0.9641 ± 0.0246	0.9744 ± 0.0014

**Table 2:** Comparison of ablation experiment results on the test set.

Fold	Model	ACC	Rec	Pre	AUC	MCC	F1	SP	AUPRC
Fold 1	ATPPred	**0.9104**	**0.8996**	0.9194	**0.9627**	**0.8210**	**0.9094**	0.9211	**0.9721**
	ATPPred (ProtBERT)	0.8047	0.8065	0.8036	0.8926	0.6093	0.8050	0.8029	0.8935
	ATPPred (Frozen ESM)	0.7903	0.7634	0.8068	0.8694	0.5815	0.7845	0.8172	0.8849
	ESM + MLP	0.8835	0.8351	**0.9246**	0.9431	0.7706	0.8776	**0.9319**	0.9571
Fold 2	ATPPred	**0.9194**	**0.8638**	0.9718	**0.9702**	**0.8439**	**0.9146**	0.9749	**0.9749**
	ATPPred (ProtBERT)	0.8262	0.8423	0.8160	0.9118	0.6527	0.8289	0.8100	0.9211
	ATPPred (Frozen ESM)	0.7760	0.6989	0.8263	0.8746	0.5586	0.7573	0.8530	0.8808
	ESM + MLP	0.8961	0.8029	**0.9868**	0.9544	0.8062	0.8854	**0.9892**	0.9646
Fold 3	ATPPred	**0.9265**	**0.8853**	0.9648	**0.9673**	**0.8560**	**0.9234**	0.9677	**0.9756**
	ATPPred (ProtBERT)	0.8208	0.7706	0.8566	0.9069	0.6448	0.8113	0.8710	0.9127
	ATPPred (Frozen ESM)	0.7796	0.7348	0.8071	0.8628	0.5614	0.7692	0.8244	0.8743
	ESM + MLP	0.8943	0.8100	**0.9741**	0.9659	0.8000	0.8845	**0.9785**	0.9734
Fold 4	ATPPred	**0.9211**	0.8674	**0.9719**	**0.9690**	**0.8472**	**0.9167**	**0.9749**	**0.9752**
	ATPPred (ProtBERT)	0.8190	**0.8710**	0.7890	0.8907	0.6415	0.8279	0.7670	0.8895
	ATPPred (Frozen ESM)	0.7814	0.6416	0.8905	0.8527	0.5861	0.7458	0.9211	0.8698
	ESM + MLP	0.8996	0.8459	0.9478	0.9516	0.8039	0.8939	0.9534	0.9661
Fold 5	ATPPred	**0.9176**	0.8530	**0.9794**	**0.9655**	**0.8422**	**0.9119**	**0.9821**	**0.9744**
	ATPPred (ProtBERT)	0.8136	0.7993	0.8229	0.8934	0.6275	0.8109	0.8280	0.8988
	ATPPred (Frozen ESM)	0.8029	0.7921	0.8095	0.8859	0.6059	0.8007	0.8136	0.8991
	ESM + MLP	0.8943	**0.9104**	0.8819	0.9543	0.7889	0.8959	0.8781	0.9639
Avg	ATPPred	**0.9190 ± 0.0058**	**0.8738 ± 0.0185**	**0.9615 ± 0.0241**	**0.9669 ± 0.0030**	**0.8421 ± 0.0129**	**0.9152 ± 0.0053**	**0.9641 ± 0.0246**	**0.9744 ± 0.0014**
	ATPPred (ProtBERT)	0.8169 ± 0.0082	0.8179 ± 0.0391	0.8176 ± 0.0253	0.8991 ± 0.0096	0.6352 ± 0.0171	0.8168 ± 0.0109	0.8158 ± 0.0380	0.9031 ± 0.0133
	ATPPred (Frozen ESM)	0.7860 ± 0.0108	0.7262 ± 0.0585	0.8280 ± 0.0358	0.8691 ± 0.0125	0.5787 ± 0.0194	0.7715 ± 0.0217	0.8459 ± 0.0448	0.8818 ± 0.0113
	ESM + MLP	0.8936 ± 0.0060	0.8409 ± 0.0427	0.9430 ± 0.0418	0.9539 ± 0.0082	0.7939 ± 0.0146	0.8875 ± 0.0075	0.9462 ± 0.0441	0.9650 ± 0.0058

Note: bold text indicates the best-performing classifier.

**Table 3:** Comparison of performance metrics between the two models.

Model	ACC	Rec	Pre	AUC	MCC	F1	SP	AUPRC
Hou et al. [30]	0.8954	0.9189	/	0.9571	0.7915	/	0.8718	/
ATPPred	**0.9640 ± 0.0143**	**0.9637 ± 0.0179**	**0.9635 ± 0.0250**	**0.9776 ± 0.0135**	**0.9277 ± 0.0292**	**0.9634 ± 0.0166**	**0.9654 ± 0.0191**	**0.9756 ± 0.0228**

Note: bold text indicates the best-performing classifier.

## Data Availability

The source code supporting this study is publicly available on GitHub at: https://github.com/ZhangHongqi215/ATPPred (accessed on 20 September 2026). The datasets analyzed and/or generated during the current study are also available in the same GitHub repository at: https://github.com/ZhangHongqi215/ATPPred/tree/main/data (accessed on 20 September 2026).

## Supplementary Materials

The following supporting information can be downloaded at: https://www.mdpi.com/article/10.3390/ijms27198566/s1.

## Abbreviations

The following abbreviations are used in this manuscript:

ATP	Adenosine 5′-triphosphate
MVK	Mevalonate kinase
ABC	ATP-binding cassette
ESM2	Evolutionary scale modeling 2
ESM	Evolutionary scale modeling
1D CNN	1-dimensional convolutional neural network
ACC	Accuracy
MCC	Matthews correlation coefficient
F1	F1 score
AUC	Area under the receiver operating characteristic curve
Rec	Recall
Pre	Precision
SP	Specificity
ROC	Receiver operating characteristic curve
PRC	Precision–Recall curve
RoPE	Rotary position embedding
