# Supplementary material for: ATPPred: Prediction of Plant ATP-Binding Proteins Using ESM2 and 1D Convolution
Source: Int J Mol Sci. 2026 Sep 25;27(19):8566. doi: 10.3390/ijms27198566 (PMC13664943; doi:10.3390/ijms27198566)
Supplement: Supplementary file 1 [file ijms-27-08566-s001.zip › ijms-4391556-supplementary.pdf]

Table S1. Evaluation results of the 10-fold cross-validation models on the test set.

Fold	ACC	Rec	Pre	AUC	MCC	F1	SP	AUPRC
Fold 1	0.9301	0.8996	0.9580	0.9691	0.8618	0.9279	0.9606	0.9750
Fold 2	0.9247	0.8817	0.9647	0.9651	0.8526	0.9213	0.9677	0.9735
Fold 3	0.9373	0.9032	0.9692	0.9802	0.8766	0.9351	0.9713	0.9833
Fold 4	0.9122	0.8423	0.9792	0.9655	0.8325	0.9056	0.9821	0.9744
Fold 5	0.9122	0.8423	0.9792	0.9672	0.8325	0.9056	0.9821	0.9746
Fold 6	0.9265	0.9032	0.9474	0.9727	0.8540	0.9248	0.9498	0.9784
Fold 7	0.9265	0.8746	0.9760	0.9682	0.8577	0.9225	0.9785	0.9762
Fold 8	0.8961	0.8100	0.9784	0.9701	0.8041	0.8863	0.9821	0.9749
Fold 9	0.9355	0.9140	0.9551	0.9678	0.8718	0.9341	0.9570	0.9771
Fold 10	0.9247	0.8996	0.9472	0.9647	0.8505	0.9228	0.9498	0.9727
Avg	0.9226 ± 0.0125	0.8770 ± 0.0345	0.9654 ± 0.0129	0.9691 ± 0.0046	0.8494 ± 0.0214	0.9186 ± 0.0151	0.9681 ± 0.0131	0.9760 ± 0.0031

Table S2. Performance evaluation of the ATPPred model with multi-round negative sampling on the test set.

Iteration	Fold	ACC	Rec	Pre	AUC	MCC	F1	SP	AUPRC
1	Fold 1	0.9104	0.8996	0.9194	0.9627	0.8210	0.9094	0.9211	0.9721
	Fold 2	0.9194	0.8638	0.9718	0.9702	0.8439	0.9146	0.9749	0.9749
	Fold 3	0.9265	0.8853	0.9648	0.9673	0.8560	0.9234	0.9677	0.9756
	Fold 4	0.9211	0.8674	0.9719	0.9690	0.8472	0.9167	0.9749	0.9752
	Fold 5	0.9176	0.8530	0.9794	0.9655	0.8422	0.9119	0.9821	0.9744
	Avg	0.9190 ± 0.0058	0.8738 ± 0.0185	0.9615 ± 0.0241	0.9669 ± 0.0030	0.8421 ± 0.0129	0.9152 ± 0.0053	0.9641 ± 0.0246	0.9744 ± 0.0014
2	Fold 1	0.9068	0.8387	0.9710	0.9588	0.8213	0.9000	0.9749	0.9670
	Fold 2	0.9014	0.8566	0.9409	0.9569	0.8061	0.8968	0.9462	0.9682
	Fold 3	0.9104	0.8674	0.9490	0.9545	0.8238	0.9064	0.9534	0.9675
	Fold 4	0.9032	0.8530	0.9482	0.9622	0.8105	0.8981	0.9534	0.9704
	Fold 5	0.9032	0.8602	0.9412	0.9579	0.8095	0.8989	0.9462	0.9655
	Avg	0.9050 ± 0.0036	0.8552 ± 0.0106	0.9501 ± 0.0123	0.9581 ± 0.0028	0.8142 ± 0.0078	0.9000 ± 0.0037	0.9548 ± 0.0118	0.9677 ± 0.0018
3	Fold 1	0.9086	0.8817	0.9318	0.9555	0.8184	0.9061	0.9355	0.9655
	Fold 2	0.8925	0.8781	0.9041	0.9580	0.7853	0.8909	0.9068	0.9668
	Fold 3	0.9032	0.9176	0.8920	0.9603	0.8068	0.9046	0.8889	0.9684
	Fold 4	0.8925	0.8674	0.9132	0.9485	0.7859	0.8897	0.9176	0.9572
	Fold 5	0.9014	0.8746	0.9242	0.9582	0.8040	0.8987	0.9283	0.9659
	Avg	0.8996 ± 0.0070	0.8839 ± 0.0196	0.9131 ± 0.0158	0.9561 ± 0.0046	0.8001 ± 0.0143	0.8980 ± 0.0076	0.9154 ± 0.0184	0.9648 ± 0.0044
4	Fold 1	0.9014	0.8530	0.9444	0.9559	0.8067	0.8964	0.9498	0.9650
	Fold 2	0.9014	0.8817	0.9179	0.9633	0.8035	0.8995	0.9211	0.9683
	Fold 3	0.9140	0.8961	0.9294	0.9672	0.8285	0.9124	0.9319	0.9719
	Fold 4	0.9140	0.8638	0.9602	0.9624	0.8322	0.9094	0.9642	0.9715
	Fold 5	0.9158	0.8889	0.9394	0.9601	0.8327	0.9134	0.9427	0.9667
	Avg	0.9093 ± 0.0073	0.8767 ± 0.0179	0.9383 ± 0.0159	0.9618 ± 0.0042	0.8207 ± 0.0144	0.9062 ± 0.0078	0.9419 ± 0.0165	0.9687 ± 0.0030
5	Fold 1	0.9014	0.8746	0.9242	0.9508	0.8040	0.8987	0.9283	0.9634
	Fold 2	0.9032	0.8566	0.9447	0.9575	0.8100	0.8985	0.9498	0.9671
	Fold 3	0.9050	0.8925	0.9154	0.9648	0.8103	0.9038	0.9176	0.9706
	Fold 4	0.8907	0.8459	0.9291	0.9533	0.7845	0.8856	0.9355	0.9647
	Fold 5	0.9050	0.8566	0.9484	0.9556	0.8139	0.9002	0.9534	0.9673
	Avg	0.9011 ± 0.0060	0.8652 ± 0.0184	0.9324 ± 0.0139	0.9564 ± 0.0053	0.8045 ± 0.0118	0.8974 ± 0.0069	0.9369 ± 0.0149	0.9666 ± 0.0028

Table S3. Performance comparison of ATPPred under different negative sampling strategies on the test set.

Negative Sampling Strategy	Fold	ACC	Rec	Pre	AUC	MCC	F1	SP	AUPRC
Length-matched	Fold 1	0.8949	0.8598	0.9246	0.9563	0.7917	0.8910	0.9299	0.9640
	Fold 2	0.8832	0.8598	0.9020	0.9533	0.7672	0.8804	0.9065	0.9614
	Fold 3	0.8738	0.8551	0.8883	0.9384	0.7482	0.8714	0.8925	0.9497
	Fold 4	0.8551	0.8692	0.8455	0.9411	0.7106	0.8571	0.8411	0.9524
	Fold 5	0.8949	0.8551	0.9289	0.9523	0.7922	0.8905	0.9346	0.9601
	Avg	0.8804 ± 0.0167	0.8598 ± 0.0058	0.8979 ± 0.0336	0.9483 ± 0.0080	0.7620 ± 0.0341	0.8781 ± 0.0142	0.9009 ± 0.0376	0.9575 ± 0.0061
Species-matched	Fold 1	0.8949	0.8514	0.9325	0.9562	0.7929	0.8902	0.9384	0.9645
	Fold 2	0.8768	0.8297	0.9160	0.9349	0.7570	0.8707	0.9239	0.9500
	Fold 3	0.9130	0.8804	0.9419	0.9683	0.8278	0.9101	0.9457	0.9743
	Fold 4	0.8859	0.8913	0.8817	0.9563	0.7718	0.8865	0.8804	0.9655
	Fold 5	0.8949	0.8514	0.9325	0.9568	0.7929	0.8902	0.9384	0.9663
	Avg	0.8931 ± 0.0134	0.8608 ± 0.0248	0.9209 ± 0.0238	0.9545 ± 0.0121	0.7885 ± 0.0267	0.8895 ± 0.0140	0.9254 ± 0.0264	0.9641 ± 0.0088

Table S4. Results at similarity thresholds ranging from 0.4 to 0.9 on the test set.

Threshold	Fold	ACC	Rec	Pre	AUC	MCC	F1	SP	AUPRC
0.4	Fold 1	0.8444	0.8222	0.8605	0.9162	0.6896	0.8409	0.8667	0.9278
	Fold 2	0.8556	0.8815	0.8380	0.9380	0.7121	0.8592	0.8296	0.9509
	Fold 3	0.8481	0.8519	0.8456	0.9235	0.6963	0.8487	0.8444	0.9388
	Fold 4	0.8556	0.7778	0.9211	0.9238	0.7199	0.8434	0.9333	0.9404
	Fold 5	0.8296	0.7778	0.8678	0.8941	0.6628	0.8203	0.8815	0.9122
	Avg	0.8467 ± 0.0107	0.8222 ± 0.0457	0.8666 ± 0.0327	0.9191 ± 0.0161	0.6961 ± 0.0222	0.8425 ± 0.0143	0.8711 ± 0.0401	0.9340 ± 0.0147
0.5	Fold 1	0.8293	0.7805	0.8649	0.9217	0.6617	0.8205	0.8780	0.9367
	Fold 2	0.8720	0.8232	0.9122	0.9330	0.7475	0.8654	0.9207	0.9472
	Fold 3	0.8415	0.7378	0.9308	0.9111	0.6981	0.8231	0.9451	0.9308
	Fold 4	0.8384	0.7317	0.9302	0.9076	0.6928	0.8191	0.9451	0.9282
	Fold 5	0.8537	0.7866	0.9085	0.9340	0.7138	0.8431	0.9207	0.9482
	Avg	0.8470 ± 0.0165	0.7720 ± 0.0378	0.9093 ± 0.0268	0.9215 ± 0.0121	0.7028 ± 0.0313	0.8342 ± 0.0199	0.9219 ± 0.0274	0.9382 ± 0.0092
0.6	Fold 1	0.8620	0.8073	0.9064	0.9164	0.7283	0.8540	0.9167	0.9335
	Fold 2	0.8568	0.8125	0.8914	0.9135	0.7164	0.8501	0.9010	0.9316
	Fold 3	0.8750	0.8073	0.9337	0.9419	0.7570	0.8659	0.9427	0.9542
	Fold 4	0.8464	0.7083	0.9784	0.9272	0.7207	0.8218	0.9844	0.9474
	Fold 5	0.8594	0.8125	0.8966	0.9161	0.7219	0.8525	0.9062	0.9354
	Avg	0.8599 ± 0.0103	0.7896 ± 0.0455	0.9213 ± 0.0358	0.9230 ± 0.0118	0.7289 ± 0.0163	0.8489 ± 0.0163	0.9302 ± 0.0343	0.9404 ± 0.0099
0.7	Fold 1	0.8801	0.8326	0.9200	0.9382	0.7636	0.8741	0.9276	0.9528
	Fold 2	0.8756	0.8190	0.9235	0.9413	0.7560	0.8681	0.9321	0.9540
	Fold 3	0.8824	0.8597	0.9005	0.9471	0.7655	0.8796	0.9050	0.9574
	Fold 4	0.8846	0.8462	0.9167	0.9412	0.7715	0.8800	0.9231	0.9560
	Fold 5	0.8801	0.8643	0.8925	0.9428	0.7606	0.8782	0.8959	0.9522
	Avg	0.8806 ± 0.0033	0.8444 ± 0.0188	0.9106 ± 0.0134	0.9421 ± 0.0032	0.7634 ± 0.0058	0.8760 ± 0.0050	0.9167 ± 0.0155	0.9545 ± 0.0022
0.8	Fold 1	0.8873	0.8589	0.9103	0.9500	0.7759	0.8838	0.9157	0.9463
	Fold 2	0.8773	0.9032	0.8582	0.9518	0.7556	0.8802	0.8514	0.9627
	Fold 3	0.8753	0.7984	0.9429	0.9595	0.7594	0.8646	0.9518	0.9655
	Fold 4	0.8913	0.8589	0.9181	0.9556	0.7843	0.8875	0.9237	0.9631
	Fold 5	0.8893	0.8306	0.9406	0.9563	0.7840	0.8822	0.9478	0.9636
	Avg	0.8841 ± 0.0073	0.8500 ± 0.0388	0.9140 ± 0.0342	0.9546 ± 0.0038	0.7718 ± 0.0136	0.8797 ± 0.0088	0.9181 ± 0.0403	0.9602 ± 0.0079
0.9	Fold 1	0.9104	0.8996	0.9194	0.9627	0.8210	0.9094	0.9211	0.9721
	Fold 2	0.9194	0.8638	0.9718	0.9702	0.8439	0.9146	0.9749	0.9749
	Fold 3	0.9265	0.8853	0.9648	0.9673	0.8560	0.9234	0.9677	0.9756
	Fold 4	0.9211	0.8674	0.9719	0.9690	0.8472	0.9167	0.9749	0.9752
	Fold 5	0.9176	0.8530	0.9794	0.9655	0.8422	0.9119	0.9821	0.9744
	Avg	0.9190 ± 0.0058	0.8738 ± 0.0185	0.9615 ± 0.0241	0.9669 ± 0.0030	0.8421 ± 0.0129	0.9152 ± 0.0053	0.9641 ± 0.0246	0.9744 ± 0.0014

Table S5. 10-fold cross-validation results of ATPPred on the Base-Data.

**Fold**	**ACC**	**Rec**	**Pre**	**AUC**	**MCC**	**F1**	**SP**	**AUPRC**
Fold 1	0.9600	0.9750	0.9398	0.9801	0.9202	0.9571	0.9474	0.9832
Fold 2	0.9714	0.9667	0.9775	0.9718	0.9429	0.9721	0.9765	0.9781
Fold 3	0.9886	0.9881	0.9881	0.9974	0.9771	0.9881	0.9890	0.9975
Fold 4	0.9543	0.9394	0.9789	0.9793	0.9085	0.9588	0.9737	0.9876
Fold 5	0.9829	0.9888	0.9778	0.9859	0.9658	0.9832	0.9767	0.9802
Fold 6	0.9543	0.9490	0.9688	0.9768	0.9077	0.9588	0.9610	0.9847
Fold 7	0.9600	0.9551	0.9659	0.9696	0.9201	0.9605	0.9651	0.9756
Fold 8	0.9543	0.9765	0.9326	0.9753	0.9096	0.9540	0.9333	0.9578
Fold 9	0.9429	0.9429	0.9167	0.9481	0.8818	0.9296	0.9429	0.9185
Fold 10	0.9714	0.9560	0.9886	0.9920	0.9434	0.9721	0.9881	0.9928
Avg	0.9640 ± 0.0143	0.9637 ± 0.0179	0.9635 ± 0.0250	0.9776 ± 0.0135	0.9277 ± 0.0292	0.9634 ± 0.0166	0.9654 ± 0.0191	0.9756 ± 0.0228

Table S6. Performance evaluation of the model under different batch size configurations on the test set.

Batch size	Fold	ACC	Rec	Pre	AUC	MCC	F1	SP	AUPRC
1	Fold 1	0.9104	0.8996	0.9194	0.9627	0.8210	0.9094	0.9211	0.9721
	Fold 2	0.9194	0.8638	0.9718	0.9702	0.8439	0.9146	0.9749	0.9749
	Fold 3	0.9265	0.8853	0.9648	0.9673	0.8560	0.9234	0.9677	0.9756
	Fold 4	0.9211	0.8674	0.9719	0.9690	0.8472	0.9167	0.9749	0.9752
	Fold 5	0.9176	0.8530	0.9794	0.9655	0.8422	0.9119	0.9821	0.9744
	Avg	0.9190 ± 0.0058	0.8738 ± 0.0185	0.9615 ± 0.0241	0.9669 ± 0.0030	0.8421 ± 0.0129	0.9152 ± 0.0053	0.9641 ± 0.0246	0.9744 ± 0.0014
2	Fold 1	0.8907	0.8566	0.9192	0.9537	0.7832	0.8868	0.9247	0.9630
	Fold 2	0.8978	0.8996	0.8964	0.9538	0.7957	0.8980	0.8961	0.9483
	Fold 3	0.9068	0.8351	0.9749	0.9708	0.8221	0.8996	0.9785	0.9764
	Fold 4	0.9086	0.8495	0.9634	0.9604	0.8230	0.9029	0.9677	0.9693
	Fold 5	0.9014	0.8602	0.9375	0.9601	0.8056	0.8972	0.9427	0.9697
	Avg	0.9011 ± 0.0072	0.8602 ± 0.0240	0.9383 ± 0.0320	0.9598 ± 0.0070	0.8059 ± 0.0171	0.8969 ± 0.0061	0.9419 ± 0.0332	0.9653 ± 0.0106
4	Fold 1	0.9014	0.8459	0.9516	0.9681	0.8079	0.8956	0.9570	0.9733
	Fold 2	0.9176	0.8889	0.9430	0.9712	0.8365	0.9151	0.9462	0.9753
	Fold 3	0.8530	0.7455	0.9498	0.9200	0.7230	0.8353	0.9606	0.9386
	Fold 4	0.9176	0.8638	0.9679	0.9666	0.8400	0.9129	0.9713	0.9735
	Fold 5	0.9176	0.8925	0.9396	0.9722	0.8362	0.9154	0.9427	0.9759
	Avg	0.9014 ± 0.0280	0.8473 ± 0.0600	0.9504 ± 0.0109	0.9596 ± 0.0223	0.8087 ± 0.0496	0.8949 ± 0.0343	0.9556 ± 0.0115	0.9673 ± 0.0161
8	Fold 1	0.8961	0.8925	0.8989	0.9589	0.7921	0.8957	0.8996	0.9651
	Fold 2	0.8548	0.7993	0.8992	0.9285	0.7141	0.8463	0.9104	0.9385
	Fold 3	0.7240	0.7312	0.7208	0.7982	0.4481	0.7260	0.7168	0.8014
	Fold 4	0.9014	0.8280	0.9706	0.9547	0.8117	0.8936	0.9749	0.9669
	Fold 5	0.8746	0.8172	0.9231	0.9306	0.7541	0.8669	0.9319	0.9457
	Avg	0.8502 ± 0.0729	0.8136 ± 0.0580	0.8825 ± 0.0950	0.9142 ± 0.0663	0.7040 ± 0.1479	0.8457 ± 0.0700	0.8867 ± 0.0993	0.9235 ± 0.0694

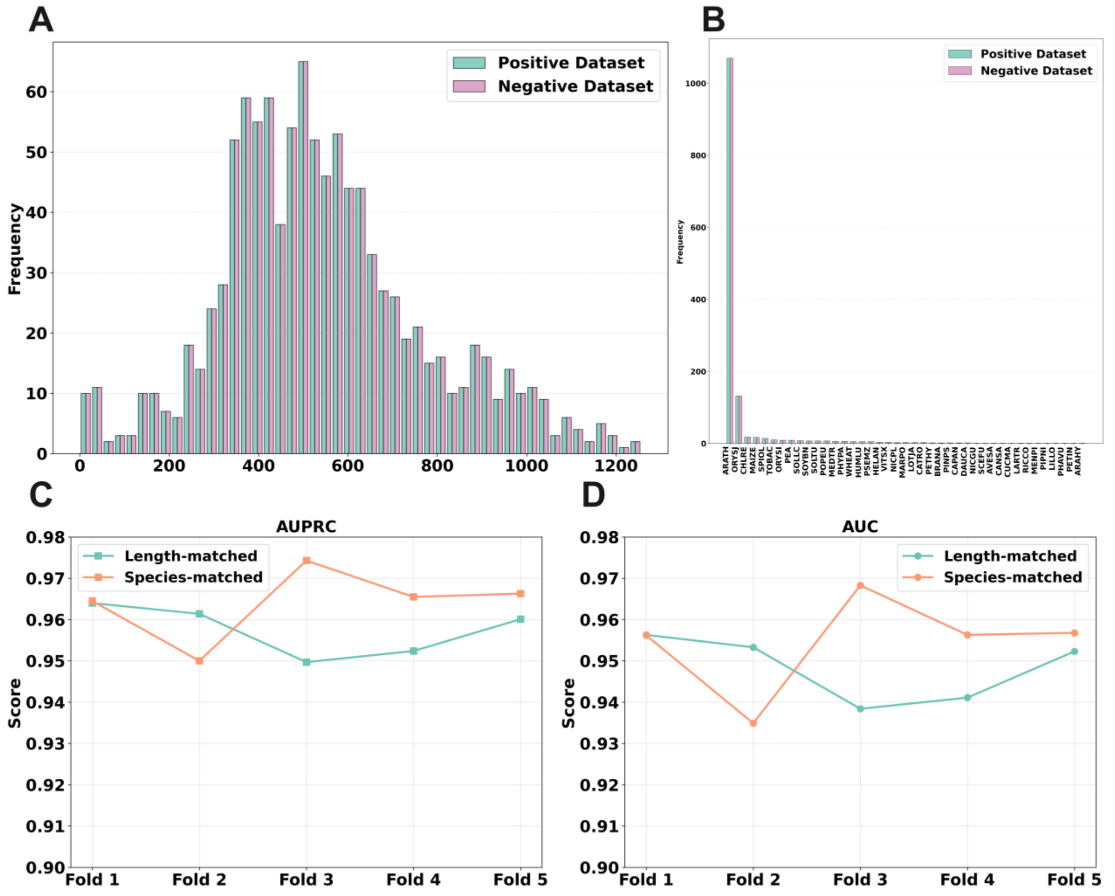

Figure S1. Performance comparison of ATPPred under different negative sampling strategies on the test set. (A) Distribution statistics for length-matched sampling. (B) Distribution statistics for species-matched sampling. (C) AUPRC results for length-matched and species-matched strategies. (D) AUC results for length-matched and species-matched strategies.

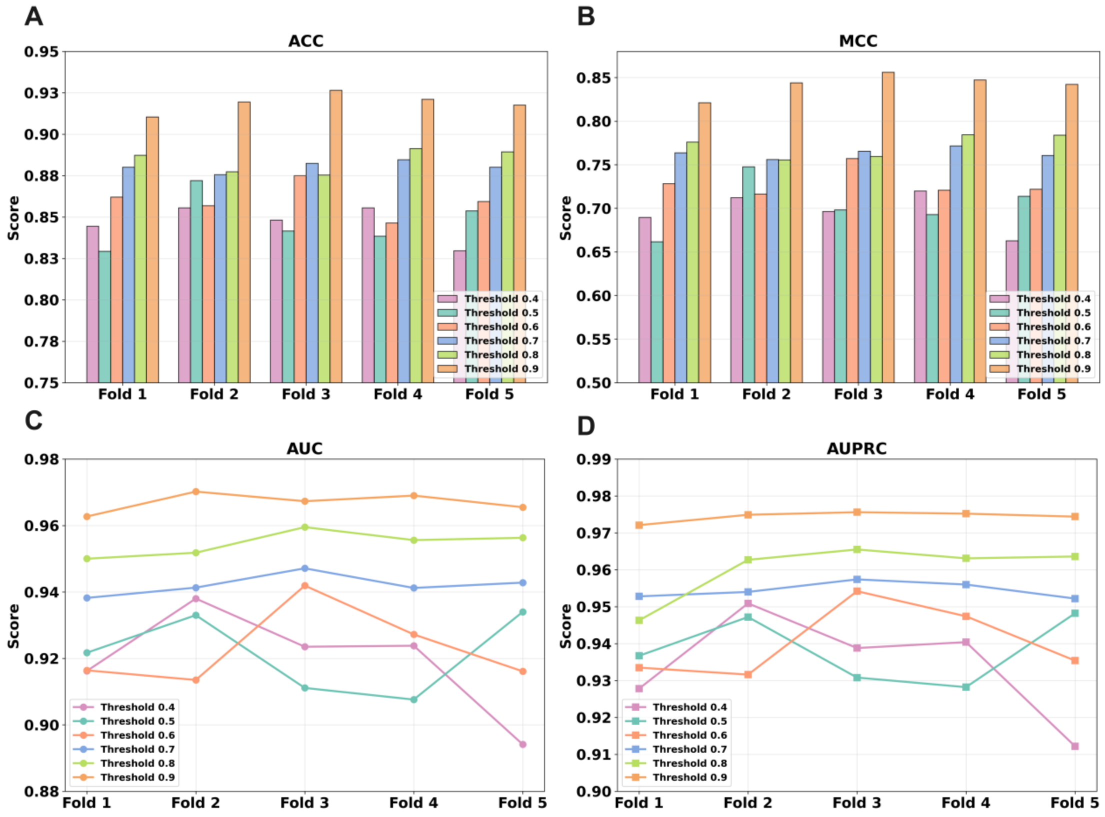

Figure S2. Performance results of ATPPred under different similarity thresholds on the test set. (A) ACC results of ATPPred at different similarity thresholds. (B) MCC results of ATPPred at different similarity thresholds. (C) AUC results of ATPPred at different similarity thresholds. (D) AUPRC results of ATPPred at different similarity thresholds.

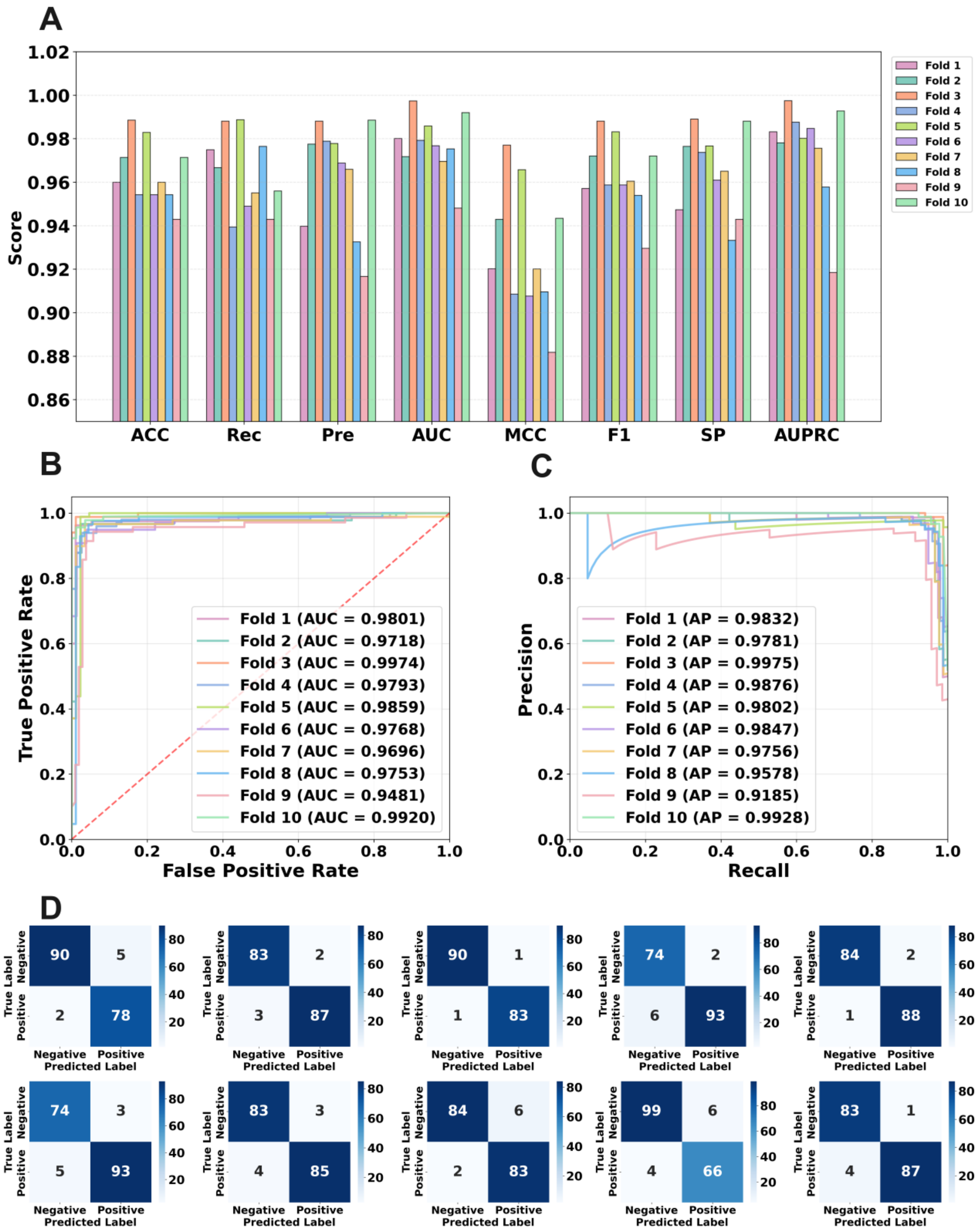

Figure S3. 10-fold cross-validation performance of ATPPred on the Base-Data benchmark dataset. (A) Evaluation metrics of ATPPred on Base-Data. (B) ROC curves from 10-fold cross-validation. (C) PRC curves from 10-fold cross-validation. (D) Confusion matrix summarizing the classification results on Base-Data.
